# Evidence for scale-dependent root-augmentation feedback and its role in halting the spread of a pantropical shrub into an endemic sedge

**DOI:** 10.1093/pnasnexus/pgac294

**Published:** 2022-12-19

**Authors:** Jamie J R Bennett, Anabele S Gomes, Michel A Ferré, Bidesh K Bera, Fabian Borghetti, Ragan M Callaway, Ehud Meron

**Affiliations:** Department of Solar Energy and Environmental Physics, Blaustein Institutes for Desert Research, Ben-Gurion University of the Negev, Sede Boqer Campus 8499000, Israel; Department of Botany, University of Brasília, Brasília 70910-900, Brazil; Department of Solar Energy and Environmental Physics, Blaustein Institutes for Desert Research, Ben-Gurion University of the Negev, Sede Boqer Campus 8499000, Israel; Department of Solar Energy and Environmental Physics, Blaustein Institutes for Desert Research, Ben-Gurion University of the Negev, Sede Boqer Campus 8499000, Israel; Department of Botany, University of Brasília, Brasília 70910-900, Brazil; Department of Wildlife Biology, University of Montana, Missoula, MT 59812, USA; Department of Solar Energy and Environmental Physics, Blaustein Institutes for Desert Research, Ben-Gurion University of the Negev, Sede Boqer Campus 8499000, Israel; Physics Department, Ben-Gurion University of the Negev, Beer Sheva 8410501, Israel

**Keywords:** vegetation pattern formation, mathematical modeling, patchy invasion, shrubland–grassland systems

## Abstract

Vegetation pattern formation is a widespread phenomenon in resource-limited environments, but the driving mechanisms are largely unconfirmed empirically. Combining results of field studies and mathematical modeling, empirical evidence for a generic pattern-formation mechanism is demonstrated with the clonal shrub *Guilandina bonduc* L. (hereafter *Guilandina*) on the Brazilian island of Trindade. The mechanism is associated with water conduction by laterally spread roots and root augmentation as the shoot grows—a crucial element in the positive feedback loop that drives spatial patterning. Assuming precipitation-dependent root–shoot relations, the model accounts for the major vegetation landscapes on Trindade Island, substantiating lateral root augmentation as the driving mechanism of *Guilandina* patterning. *Guilandina* expands into surrounding communities dominated by the Trindade endemic, *Cyperus atlanticus* Hemsl. (hereafter *Cyperus*). It appears to do so by decreasing the water potential in soils below *Cyperus* through its dense lateral roots, leaving behind a patchy *Guilandina*-only landscape. We use this system to highlight a novel form of invasion, likely to apply to many other systems where the invasive species is pattern-forming. Depending on the level of water stress, the invasion can take two distinct forms: (i) a complete invasion at low stress that culminates in a patchy *Guilandina*-only landscape through a spot-replication process, and (ii) an incomplete invasion at high stress that begins but does not spread, forming isolated *Guilandina* spots of fixed size, surrounded by bare-soil halos, in an otherwise uniform *Cyperus* grassland. Thus, drier climates may act selectively on pattern-forming invasive species, imposing incomplete invasion and reducing the negative effects on native species.

Significance Statement Understanding the mechanisms by which water-limited vegetation self-organizes in spatial patterns is highly significant in the current era of climate change, as spatial patterning improves the resilience of ecosystems to drier climates. However, empirical confirmation of theoretically proposed mechanisms hardly exists, and the implications of vegetation patterning to other ecological processes of high concern, such as species invasion, have eluded consideration. We address both problems by studying a model of a shrubland–grassland system on Trindade Island, where a pantropical shrub, *Guilandina bonduc* L., displaces an endemic sedge, *Cyperus atlanticus* Hemsl., via spatial patterning. We provide evidence for a basic mechanism of vegetation patterning associated with lateral root spread and predict that spatial patterning under severe water stress can halt the shrub’s invasion.

## Introduction

Ecosystems in resource-limited environments often respond to resource scarcity by self-organizing in spatial patterns of biomass and resources (1). A much studied context of spatial self-organization in ecology is water-limited landscapes, where a variety of regular and irregular vegetation patterns have been observed, including nearly periodic gap, stripe, and spot patterns (2). Model studies explain the emergence of such patterns from uniform vegetation in terms of a positive feedback loop between vegetation growth in incidental patches of denser vegetation and net water transport toward these patches from surrounding areas of sparser vegetation (3, 4). Vegetation growth increases the amount of water drawn from the surrounding areas, which, in turn, accelerates growth further. This feedback loop is scale-dependent in the sense that it combines activated vegetation growth at short scales and inhibited growth at longer scales, thereby amplifying small nonuniform perturbations to form periodic patterns (1, 5).

The most studied case of water transport is overland water flow as a result of increased water infiltration rates in denser vegetation patches (6, 7). Increased infiltration has been attributed to factors such as higher soil porosity due to denser roots and lower density of soil crusts (8, 9). Another mechanism of water transport, increasingly discussed in recent years, is lateral soil-water diffusion in loose top soil layers, such as sandy soils or gravel (10–13). In that case, the water transport is associated with the development of soil-water gradients as a result of higher transpiration in denser vegetation patches.

Another water-transport mechanism is water conduction by laterally extended roots. In this case, enhanced water transport toward patches of denser vegetation has been associated with the positive correlation between shoot and root growth; as plants grow, their roots extend in the lateral directions and take up more water from the surrounding areas (14, 15). Unlike the scale-dependent feedback associated with overland water flow and soil-water diffusion, that associated with water conduction by lateral roots—the root-augmentation feedback—has hardly been studied empirically, despite its generic nature (16–18).

Self-organizing vegetation patterns have been extensively studied (1, 19–23), but far fewer studies have addressed the implications for community dynamics  (24–28). Lacking, in particular, are studies of range expansion or invasion, in which the target plant species is capable of self-organization in spatial patterns (29), and questions addressing the effects of spatial self-organization on these processes have not been explored.

**Fig. 1.**
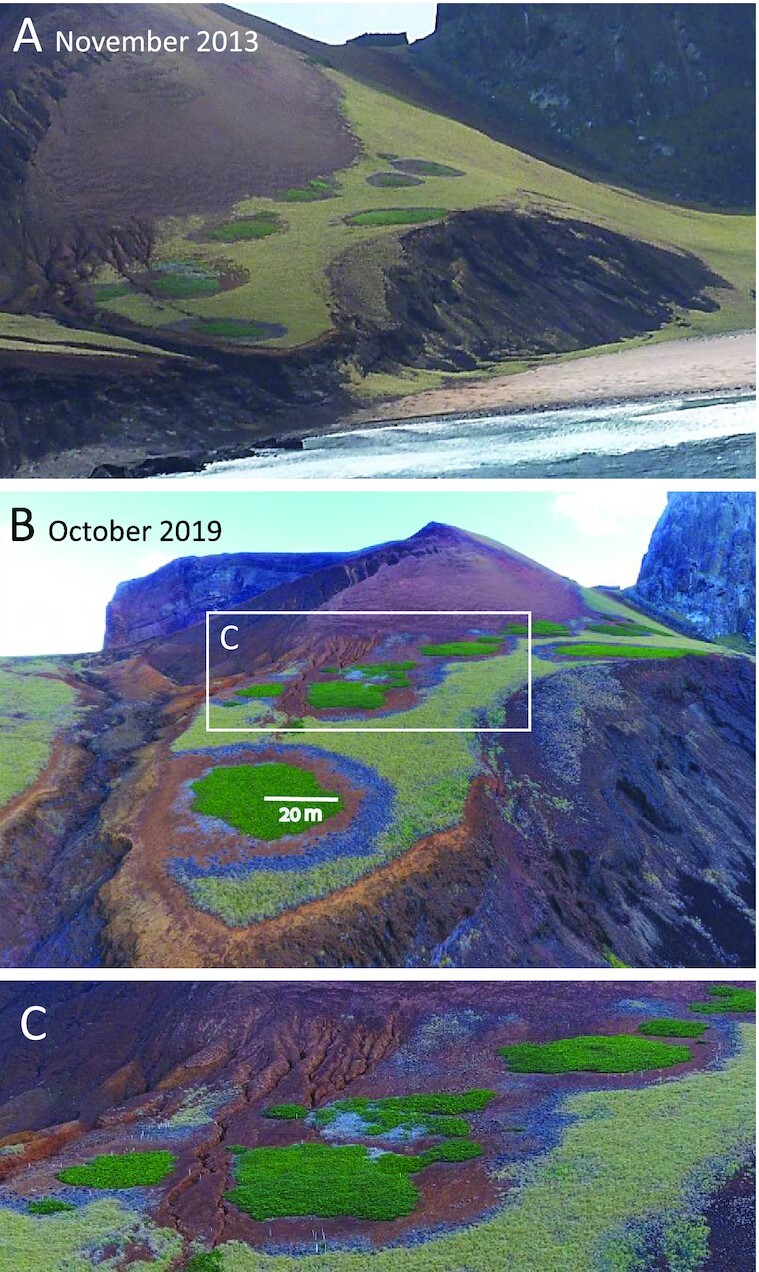
Patchy invasion at low altitudes on Trindade Island. (A) An image from 2013 November, showing spots of *Guilandina* surrounded by halos of bare soil and dead *Cyperus* isolated by a matrix of uniform *Cyperus*. (B) An image from 2019 October, showing radially expanded *Guilandina* spots and their halos, and gradual displacement of *Cyperus*. (C) A blow-up of an area of advanced invasion in image (B), where *Guilandina* spots have completely displaced *Cyperus*.

An interesting candidate system for both demonstrating the root-augmentation feedback and studying the impact of spatial self-organization on range expansion or invasion is Trindade Island, where *Guilandina bonduc* L. (hereafter *Guilandina*), a clonal pantropical shrub, is found to displace the endemic sedge *Cyperus atlanticus* Hemsl. (hereafter *Cyperus*), leaving behind a *Guilandina*-only patchy landscape, as indicated by Fig. 1. To our knowledge, *Guilandina* is native to Trindade, and thus not an “exotic invader”. However, the mechanism by which it rapidly expands after the removal of livestock and its patchy displacement of *Cyperus* may well apply to ecosystems where patchy displacement of a native species is driven by exotic invaders (30). We, therefore, refer to this form of displacement as “patchy invasion”. Aerial images of Trindade Island, as shown in Fig. 1, suggest that the invasion is patchy (31, 32), leaving behind a self-organized patterned state of *Guilandina* spots surrounded by halos of bare soil and dead *Cyperus*.

In this paper, we use the Trindade *Guilandina*–*Cyperus* ecosystem as a case study for demonstrating the hypothesized root-augmentation feedback in a real ecosystem and for highlighting the role of spatial patterning in invasion dynamics. We accomplish this goal by combining empirical observations with mathematical modeling of a woody–herbaceous system that captures the root-augmentation feedback as the dominant mechanism of spatial self-organization.

## Results

### Evidence for a root-augmentation feedback

The rationale behind the evidence we provide for the root-augmentation feedback as the major driver of *Guilandina*’s patterning is as follows. We use empirical observations of abiotic and biotic factors in Trindade Island, such as water as the growth-limiting resource, soil characteristics, *Guilandina*’s lateral root spread, clonal reproduction, and others, to construct the model. We then use the model to study the dynamics of *Guilandina*’s patterning at the single-patch scale and at the landscape scale, and confront model results with empirical observations at different sites of Trindade Island. A strong agreement between model predictions and empirical observations can provide compelling evidence for the dominant role of the root-augmentation feedback in *Guilandina*’s patterning.

#### The Trindade ecosystem

Trindade is a volcanic, tropical island in the Atlantic Ocean, lying roughly 1,200  km east of the Brazilian coast. The climate of Trindade Island is atypical of climates in which water-based vegetation patterning is expected. The island has a tropical climate without a true dry season (33), with average annual rainfall of 1476 mm at low altitudes (National Oceanographic Data Bank of the Brazilian Navy) and increased rainfall rates at higher altitudes. However, because of warm annual average temperatures (25.3^○^C), the coarse volcanic soils, and *Guilandina*’s dense lateral roots, the water potentials in the top-soil layers at low-elevation bare-soil areas can be below −3.0 MPa (34), and, thus, highly limiting.

*Guilandinabonduc*, synonymous with *Caesalpinia bonducella*, is in a pantropical genus of lianas and scandent shrubs characterized by unisexual flowers and few-seeded, oval-shaped dehiscent fruits, and are often armed with rigid trichomes or prickles. The seeds are hard and globose and adapted for long-distance oceanic dispersal by flotation (35). The first recording of *Guilandina* in Trindade Island was in 1916  (36). On Trindade, *Guilandina* generally grows as a short, spreading shrub, with clones expanding laterally in exceptionally circular patterns, and new clones appearing a few tens of meters from apparent parent plants. We observed young germinants from seeds on Trindade, based on the presence of cotyledons, but our observations in the field indicate vegetative reproduction of new individuals is far more common.

The spatial expansion of *Guilandina* and concomitant spatial patterning at low elevation does not appear to be driven by plant–soil feedback or allelopathy, based on plants grown experimentally in soils collected under and outside of *Guilandina* patches (34).

#### Patch-scale observations

**Fig. 2.**
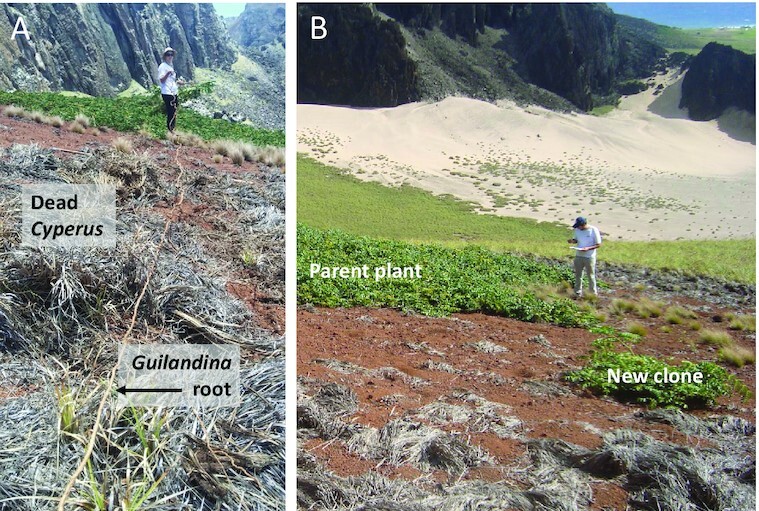
Two characteristic aspects of *Guilandina* at the low-altitude sites. (A) Lateral root extension far beyond the canopy. The root was dug up from a soil depth of 6 cm and is about 8 m long. It starts at the *Guilandina* patch in the upper part of the image and extends through a bare-soil area and an area of the dead *Cyperus*. (B) Distant sprouting of a *Guilandina* clone from a laterally extended root of the parent plant. The clone is located in the halo of the parent plant, but has sprouted at an earlier stage of the invasion process, possibly when the sprout was still in a *Cyperus*-covered area where the water potential is higher.

Four sites in Trindade Island have been studied, one at a high altitude (site 1, circa 600  m) and three at a low altitude (sites 2 to 4, circa 60  to 100 m), as illustrated by Fig. S1 in the Supplementary Material. In the absence of *Guilandina, Cyperus* forms nearly uniform vegetation coverage, with a few other grasses and forbs mixed in. The form of *Guilandina* patches depends on elevation. At low elevations (sites 2 to 4), *Guilandina* forms spot-like patches consisting of circular aboveground biomass distributions surrounded by bare-soil halos (Fig. 1). Underneath the halos of bare soil, dense *Guilandina* roots are found, as shown in Fig. S2 in Supplementary Material. Fig. 2A shows an example of an exposed root extending throughout the bare-soil halo.

Although the low-altitude sites feature moderately sloped terrains, the round shape of isolated *Guilandina* patches suggests that runoff does not play a dominant role; otherwise, we would expect to observe elongated, arc-like patches, oriented perpendicular to the slope (37). Another indication for this is the absence of dead plants at the lower lying edges of *Guilandina* patches, which would indicate runoff loss to lower vegetation patches and migration uphill (2). The absence of significant runoff may be attributed to high infiltration rates of surface water into the soil because of the coarse nature of the volcanic soil and the increased soil porosity by the presence of roots in all areas, including the bare-soil halos. The bare-soil halos are surrounded by dead-*Cyperus* halos of different widths, indicating invasion processes have recently occurred or are still occurring.

Measurements of soil-water potentials indicate much lower values in the bare-soil halos than in either the *Cyperus* or the *Guilandina* zones (indicating lower water availability), as indicated by Fig. S3 in Supplementary Material. The lowest single soil-water potential measurement was also in a bare-soil halo, −4.8 MPa.

At high elevations, *Guilandina* does not form distinct spatial patches; it is much more intermixed with *Cyperus*, and soil cores show that *Guilandina* roots do not expand into surrounding vegetation as they do at lower elevations [see Supplementary Material and Fig. S2, and Ref. (34)], presumably because of much higher precipitation and soil depth.

Our field observations found that *Guilandina*’s clones in the low-altitude sites sprout at several distances along the root system from the mother plant, producing new individuals at different points of the expanding roots. Fig. 2B shows an example of such a clone. This pattern of clonal expansion is presumably via root suckers (38), as observed in other *Caesalpinia* species (39). The long-distance clonal expansion is likely a result of the low water potential in the bare-soil halos that surround *Guilandina* patches and the higher potential beyond these halos, as shown by our measurements (see Fig. S3 in Supplementary Material).

#### Landscape-scale observations

**Fig. 3.**
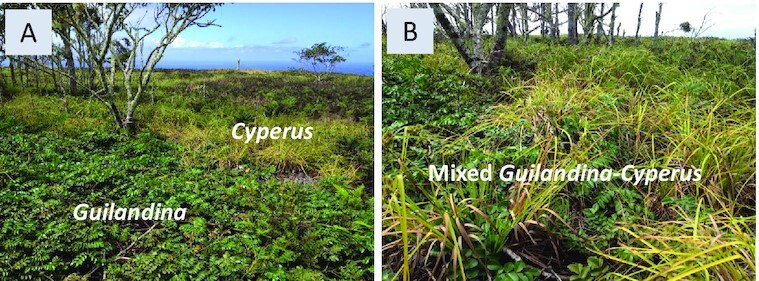
Spatial patterns at high altitudes. (A) Adjacent *Guilandina*-only and *Cyperus*-only patches. (B) Mixed *Guilandina–Cyperus* patch. In both cases, the land is fully covered by vegetation apart from sporadic small bare-soil patches.

Landscape-scale patterns depend on water availability. At high altitudes where water availability is high, full vegetation coverage is typically observed. The vegetation often forms mosaics of *Guilandina* patches, *Cyperus* patches, and mixed *Guilandina–Cyperus* patches. Fig. 3 shows examples of such patches. There is no indication of *Guilandina*’s self-organization in spatial patterns devoid of *Cyperus*. At low altitudes, isolated *Guilandina* spots surrounded by bare-soil halos in an otherwise uniform *Cyperus* grassland, or areas of nearly regular *Guilandina* spot patterns in otherwise bare soil devoid of *Cyperus*, are common (Fig. 1). The spots in these areas are of a comparable size and distance to their neighbors, with no indications of external heterogeneities that might dictate them. A spatial statistical analysis, such as calculating pair-correlation functions (40, 41), is not possible because of the insufficient number of spots for such an analysis.

#### Modeling the Trindade ecosystem

We use a continuum modeling approach (42), where all state variables are continuous functions of space and time, that solve a system of integro-partial differential equations for four state variables: aboveground biomasses of *Guilandina, B*_1_, and *Cyperus, B*_2_, soil-water content, *W*, and surface water *H*, all in units of kg/m^2^ (43). In constructing the model, we make use of three elements of the Trindade ecosystems: (i) vegetation growth is limited by water availability, (ii) *Guilandina* can develop long lateral roots, and (iii) *Guilandina* patches expand by clonal growth with nonlocal sprouting of new clones. The first element motivates the use of the model platform developed in (43) (see also  (3)) for vegetation pattern formation in water-limited systems. The second element guides us to include nonlocal root-kernel terms that account for water conduction by laterally spread roots and capture the root-augmentation feedback of *Guilandina*. The third element guides us to include a nonlocal clonal-growth kernel to describe sprouting of new *Guilandina* clones at a distance. In addition, we make use of the apparent absence of significant runoff to simplify the model by eliminating overland water dynamics, i.e., the equation for the surface water variable *H*. We verified that this simplification does not affect the results we report here, as long as the infiltration rates in bare soil and in vegetation patches are comparable and high enough relative to the rates of water uptake by the plants’ roots and biomass growth. We refer the reader to the "Materials and Methods" section for a full description of the model.

We distinguish between high- and low-altitude sites in Trindade Island by controlling two main parameters: the precipitation (mean-annual-rainfall) rate, *P*, and the lateral root extension per unit aboveground biomass, *E*—a measure for the plant’s investment in growing laterally extended roots relative to the investment in growing shoots. The mean annual rainfall at a low altitude is assumed to be in the range of 1250 to 1500 mm/y, and to increase with altitude. Exact values at high altitudes are not known, since only one weather station exists on the island; we assume a precipitation range of 1500  to 1750 mm/y. Large (small) *E* values represent *Guilandina*’s phenotypes with long (short) lateral roots in low (high) altitudes where soil-water availability is low (high). Differences between the low-altitude sites 2 to 4 can still be captured by varying the precipitation rate within the aforementioned range.

#### Model analysis

**Fig. 4.**
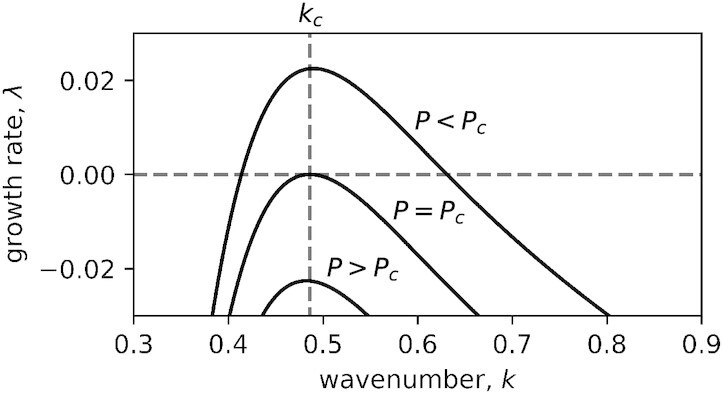
Growth rates of periodic perturbations about the uniform Guilandina-only state. Above a critical precipitation threshold, *P_c_*, perturbations of any wavenumber *k* (or wavelength 2π/*k*) decay to zero, implying stability of the uniform Guilandina-only state to any sufficiently small perturbation. As this threshold is traversed, by decreasing the precipitation rate *P*, perturbations of wavenumbers *k_c_* and close to it begin to grow, leading to the destabilization of the uniform Guilandina-only state and the development of periodic Guilandina-only patterns.

The model has four spatially uniform stationary states: a bare-soil state devoid of any vegetation (BS), a *Guilandina*-only state (GU), a *Cyperus*-only state (CU), and a mixed *Guilandina–Cyperus* state (MU). Their existence and stability properties depend on model parameters; in particular, the parameters we use to distinguish between low- and high-altitude sites are *P* (precipitation rate) and *E* (lateral root extension). The possible existence of a nonuniform stationary (Turing) instability, induced by the root-augmentation feedback, can be studied using linear stability analysis (22) of the uniform *Guilandina*-only state. Such an analysis yields a dispersion relation, *σ* = *σ*(*k; E, P*), which provides information about the growth rates, $\lambda =\operatorname{Re}(\sigma )$, of periodic perturbations of wavenumber *k* (wavelength 2π/*k*) for various values of *P* and *E*. The existence of a nonuniform stationary instability, for a given value of *E*, can be proven by identifying the critical wavenumber and precipitation values, *k_c_* and *P_c_*, at which the conditions *σ* = λ (i.e., *σ* is real valued), λ = 0, *d*λ/*dk* = 0, and *d*^2^λ/*dk*^2^ < 0 are satisfied. These conditions guarantee that as *P* is decreased below a critical value, *P_c_*, a periodic mode of a critical wavenumber, *k_c_* > 0, begins to grow monotonically in time, as shown in Fig. 4. The growth of this mode results in the formation of *Guilandina*-only stationary periodic patterns (GP). A similar analysis of the mixed *Guilandina–Cyperus* state (MU) yields the threshold at which the mixed state goes through a Turing instability to mixed *Guilandina–Cyperus* stationary periodic patterns (MP).

**Fig. 5.**
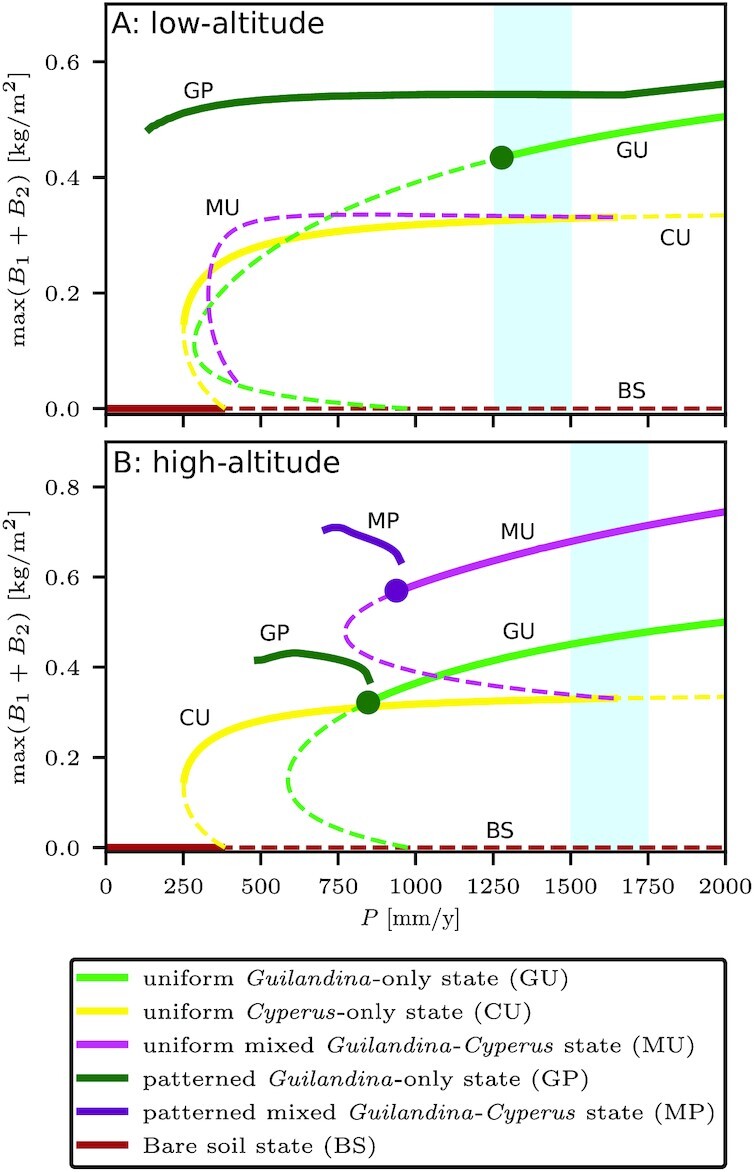
Partial bifurcation diagrams of low and high altitudes showing stationary solutions. At low altitudes (A), the lateral extent of the roots is large (*E*_1_ = 16 [m^2^/kg]), while at high altitudes (B), it is small (*E*_1_ = 5 [m^2^/kg]). In both cases, solution branches are shown for a wide precipitation range of 0 to 2000 mm/y, but the actual ranges for the two altitudes are much smaller and are estimated by the blue shaded regions. Solid (dashed) lines indicate stable (unstable) solutions. Large circles represent nonuniform stationary instabilities, from which patterned solution branches bifurcate. Though a patterned *Cyperus*-only branch exists, its range in *P* is too small to be plotted on this scale.

**Fig. 6.**
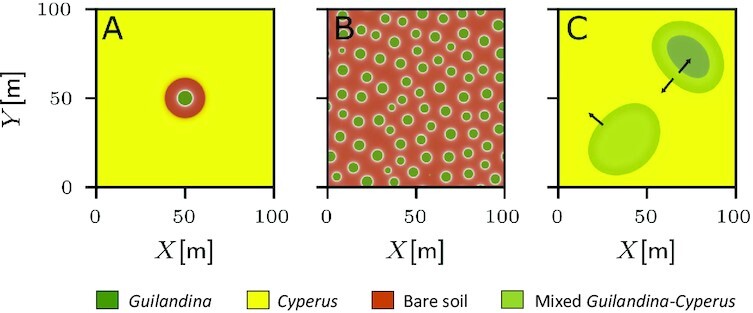
Model simulations showing spatial patterns at low and high altitudes. (A) A stationary *Guilandina* spot in a uniform *Cyperus* grassland at low altitudes. (B) A nearly stationary *Guilandina* pattern at low altitudes. (C) Growing mixed *Guilandina–Cyperus* patches in a *Cyperus* grassland at high altitudes in a tristability range of uniform *Guilandina*, uniform *Cyperus*, and uniform mixed *Guilandina–Cyperus* states. The arrows denote the directions in which state boundaries move. Parameters: (A), (B) *P* = 1000 [mm/y], *E*_1_ = 16 [m^2^/kg], $S_{D_1}=0.5\,[{\rm m}]$; (C) *P* = 1600 [mm/y], *E*_1_ = 5 [m^2^/kg], $S_{D_1}=0.01\,[{\rm m}]$. Other parameters are specified in Table 1.

The existence and stability ranges of the various system states at low and high altitudes for 1D systems are summarized in the bifurcation diagrams shown in Fig. 5. According to the bifurcation diagram for low altitudes (Fig. 5A), where roots are laterally extended, there is a wide precipitation range where the uniform *Cyperus* state (CU) and periodic *Guilandina* pattern (GP) are alternative stable states. In this range, solutions describing isolated *Guilandina* spots in an otherwise uniform *Cyperus* grassland are possible (22, 43). Indeed, 2D model simulations confirm the existence of stable isolated spot solutions, as shown in Fig. 6A. These model results, based on the assumption of the root-augmentation feedback as the dominant pattern-forming feedback at low altitudes (high *E* values), are in accord with empirical low-altitude observations of isolated circular *Guilandina* patches surrounded bybare-soil halos in an otherwise uniform *Cyperus* grassland. These model halos should be interpreted as including the dead-*Cyperus* halos in the low-altitude sites as dead vegetation is counted as zero (live) biomass. The model also predicts the possible existence of periodic *Guilandina*-only patterns (dark green solution branch in Fig. 5A), as indicated by the 2D simulation result shown in Fig. 6B. These patterns coexist with the single-spot pattern shown in Fig. 6A and were obtained using different initial conditions. The periodic *Guilandina*-only patterns predicted by the model are consistent with the observed patterns shown in Fig. 1C.

According to the bifurcation diagram for high altitudes (Fig. 5B), where roots are laterally confined, a wide tri-stability range of all three uniform states, GU, CU, and MU, exists. In this tri-stability range, *Guilandina* patches or mixed *Guilandina–Cyperus* patches in a *Cyperus*-only grassland, fully covering the soil, are among the possible states, which is consistent with Fig. 6C. These states represent long transients, as the patch boundaries (fronts) are not stationary in general (44) (see arrows in Fig. 6C). The transient dynamics eventually converge to one of the three possible uniform states, depending on the precipitation value. Periodic *Guilandina*-only patterns, as found at low altitudes, are not found at high latitudes because of the small lateral extent of the roots. An interesting numerical finding is that the boundary between *Guilandina* and *Cyperus* patches appears unstable, as it encourages simultaneous growth of the two species to form an intermediate patch of the mixed *Guilandina–Cyperus* state that grows at the expense of the original two patches (see arrows in Fig. 6C).

#### Summary of evidence for a root-augmentation feedback

The patch-scale empirical observations suggest that out of the three scale-dependent feedback that the model captures, only the root-augmentation feedback is relevant to the Trindade ecosystem, and only at low altitudes. Using parameters that reflect that situation, we found that the model reproduces the main landscape-scale observations at the two altitudes: (i) at low altitudes, sparse *Guilandina* spots surrounded by bare-soil halos in an otherwise uniform *Cyperus* grassland, as well as areas of dense *Guilandina* spots in bare soil—devoid of *Cyperus* (compare Figs. 1 and 6A and B); and (ii) at high altitudes, full vegetation coverage by *a Cyperus* grassland with *Guilandina* patches or mixed *Guilandina–Cyperus* patches of various sizes and shapes (compare Figs. 3 and 6C).

### Patchy invasion

Ecological invasions occur when species establish themselves in native ranges of other species outside their normal range. The local establishment of an invasive species consists of two distinct phases. The first involves the local growth of the invasive species from low densities, outcompeting the native species, to form an initially small invasive-species patch or spot. The second phase involves the spatial spread of this patch into the rest of the native range, often referred to as “geographical spread” (45). Several forms of geographic spread can be distinguished, including a continuous spread by means of a propagating front that bounds a simply-connected domain of the invasive species, and discontinuous spread involving the recruitment of new patches at a distance, through long-distance seed dispersal or clonal sprouting. The latter has been referred to as “patchy invasion” (46, 47). Patchy invasion may not necessarily result in a patchy state of the invasive species, as the patches that form may grow and merge into ever bigger patches until a spatially uniform state results. This is the case, for example, with the cordgrass *Spartina alterniflora* Loisel, which grows on intertidal mudflats. Each recruit germinates from a single seed and grows rhizomatously into a circular patch. These patches, initially isolated from one another, expand and eventually coalesce to form a continuous meadow (48). The invasion of *Guilandina* is also patchy, through long-distance clonal sprouting, but unlike *S. alterniflora*, it does form a patchy state after displacing *Cyperus*.

The capability of *Guilandina* to form spatial patterns has an important effect on the invasion process; the second phase of spatial spread can be of two types, incomplete invasion, resulting in spatial coexistence of the two species, *Guilandina* and *Cyperus*, and *complete invasion*, resulting in total exclusion of *Cyperus*. This is unlike the case of invasive species that are incapable of forming patterns, such as *S. alterniflora*; in this case, once the spatial spread begins, it continues all the way to exclusion. This intricate behavior of pattern-forming invasive species is rooted in the pattern-formation phenomenon of spot replication, observed experimentally in various contexts, including chemical reactions (49), gas-discharge systems (50), and vibrated dense suspensions (51), and studied theoretically using various mathematical models (52–55). Spot replication occurs in a bistability range of uniform and patterned states where solutions representing a single spot, or a bunch of isolated spots, exist. It is triggered by an instability of the single-spot solution and convergence to a coexisting stable periodic-pattern solution.

**Fig. 7.**
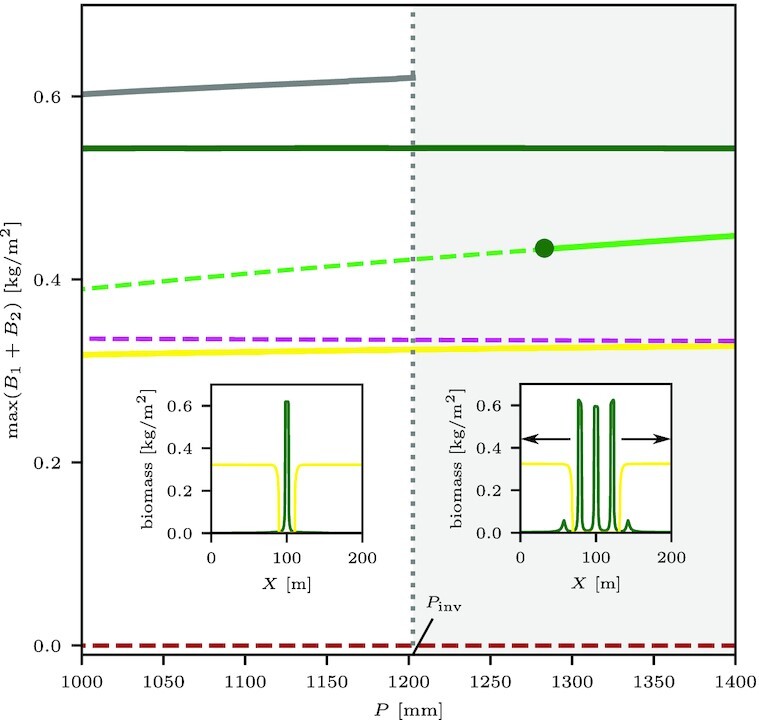
Threshold of spot replication. Close-up of the 1D bifurcation diagram in Fig. 5 showing the bistability range of uniform *Cyperus* (yellow line) and *Guilandina* (dark green line) patterns. Within this range, a stable stationary single *Guilandina*-spot solution exists for *P* < *P*_inv_ ≈ 1202.6 [mm/y] (gray line). Beyond this threshold, a single *Guilandina* spot initiates the sprouting of new spots, as shown in the two insets, triggering spot-replication dynamics.

**Fig. 8.**
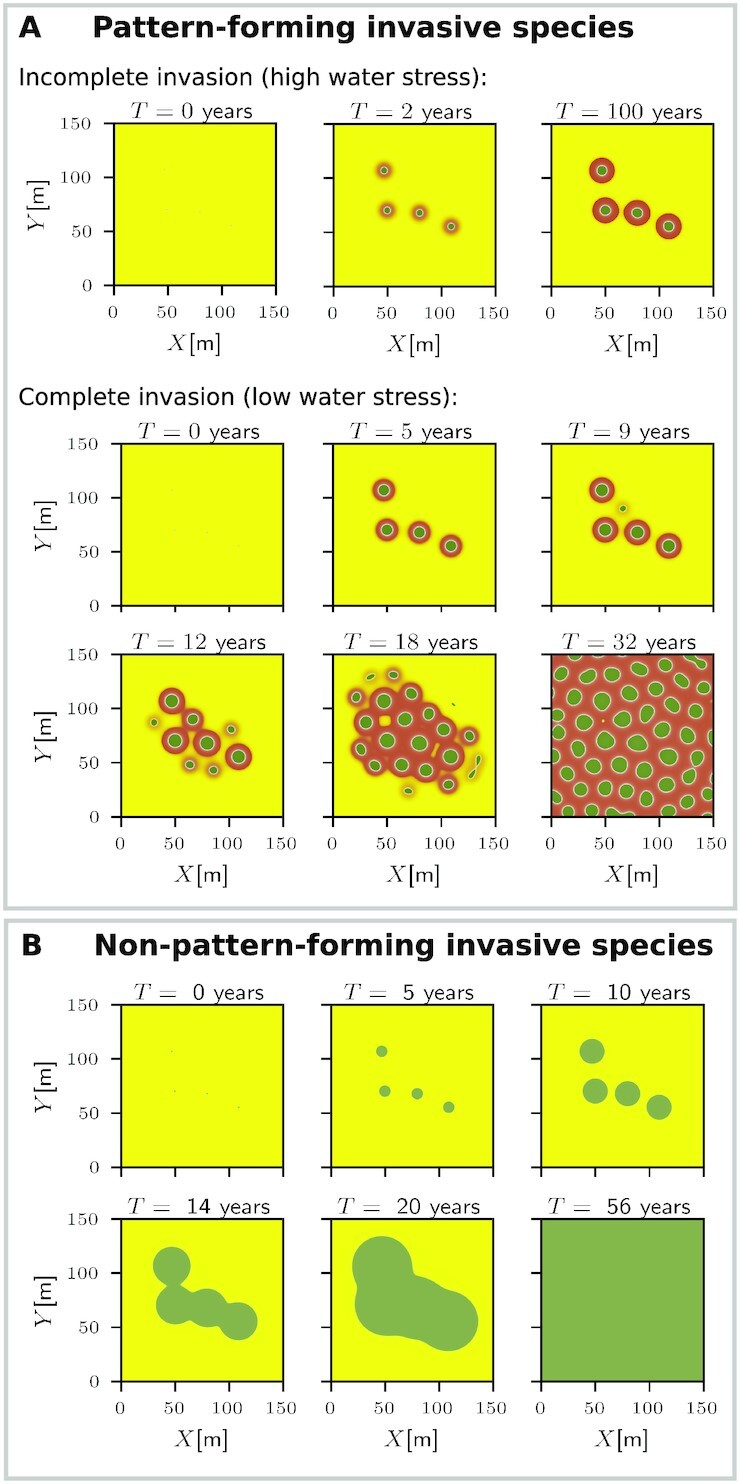
Snapshots of model simulations showing invasion dynamics by pattern-forming and non-pattern-forming species. (A) The invasion dynamics of a pattern-forming species take two forms. At sufficiently low precipitation, *P* = 1000 [mm/y] < *P*_inv_ (high water stress), the invasion is incomplete; initially, small *Guilandina* spots expand, develop halos, and converge to asymptotic spots of fixed size. At higher precipitation, *P* = 1400 [mm/y] > *P*_inv_ (low water stress), the invasion is complete; the same initial conditions develop into a *Guilandina* spot pattern devoid of *Cyperus* through a process of spot replication. (B) Invasion dynamics of a non-pattern-forming species in a bistability range of uniform native and invasive states take a single form. Once invasive-species spots begin to expand, they keep expanding and coalesce with neighbor spots to form a uniform invasive species state. Initial conditions in (A) and (B) are identical: Four very small *Guilandina*-only spots were added to a uniform *Cyperus*-only background state. Parameters for all panels are given in Table 1, except for the changed parameters in (B). The most significant change is a sharp decrease of the root-augmentation parameter *E*_1_ from 16 to 2 [m^2^/kg], a value for which the root-augmentation feedback is too weak to produce patterns. Other parameters have been adjusted as follows: *P* = 1400 [mm/y], *K*_1_ = 1.75 [kg/m^2^], *M*_1_ = *M*_2_ = 9.05 [1/y], Λ_1_ = Λ_2_ = 0.08 [(1/mm)/y], Γ_1_ = 5 [(m^2^/kg)/y], $S_{G_1}=0.01[{\rm m}]$, $S_{D_1}=0.1[{\rm m}]$, $W_2^{*} = 0.5[{\rm kg/m^2}]$, and *D_W_* = 1 [m^2^/y].

In the present case, the uniform state is formed by *Cyperus* and the patterned state by *Guilandina*. As shown in the bifurcation diagram for 1D solutions in Fig. 5, the two states form a very wide bistability range along the precipitation axis. The single-spot solution corresponds to an isolated *Guilanidina* spot in an otherwise uniform *Cyperus* grassland. The stable part of its solution branch is shown by the gray line in the 1D bifurcation diagram of Fig. 7. That stable part does not extend through the entire bistability range of uniform *Cyperus* and patterned *Guilanidina* states; beyond a precipitation threshold *P* = *P*_inv_, the solution becomes unstable or ceases to exist (shaded area in Fig. 7). The existence of the threshold *P*_inv_ (in 2D curvature corrections should be considered) is highly significant. Below the threshold, small *Guilanidina* spots expand in time, form halos of bare soil, and approach an asymptotic fixed size, but further geographical spread does not occur, as shown by the 2D simulations in the top part of Fig. 8A (see also Movie M1 in Supporting Material); the spots are incapable of triggering the emergence of new spots in their neighborhood, and, consequently, the *Cyperus* species is not excluded. By contrast, above that threshold, *Guilanidina* spots do trigger the formation of new spots in their neighborhoods, leading to complete invasion and total exclusion of *Cyperus*, as shown by the snapshots in the bottom part of Fig.8A (see also Movie M2 in Supporting Material).

This invasion form by pattern-forming species should be contrasted with the invasion by a non-pattern-forming species, where no threshold, *P* = *P*_inv_, for geographic spread exists; once invasive-species spots are formed and begin to expand, they eventually lead to complete exclusion of the native species, as shown in Fig. 8B. The intermediate form of incomplete invasion and asymptotic species coexistence does not exist in this case as there is no stable single-spot state.

## Discussion

The evidence we present here for root-augmentation feedback consists of three elements: (i) empirical findings at the single-plant level that are used to motivate a mathematical model of vegetation pattern formation where water conduction by laterally spread roots is the dominant water transport mechanism; (ii) model studies that upscale that information to the level of a fully developed patch-up to the landscape level of many patches; and (iii) confrontation of model predictions with empirical observations at both levels at different sites. The strong agreement between predicted and observed patch-level and landscape-level behaviors provides compelling evidence for the root-augmentation feedback as the driving pattern formation mechanism in the Trindade ecosystem.

More generally, the root-augmentation feedback is likely to play a dominant role in water-limited ecosystems that satisfy the following two conditions: (i) plant species having lateral roots that extend well beyond their canopies (56); and (ii) soil types that are loose enough to allow the infiltration of surface water into bare soil and form water reservoirs within the reach of laterally extended roots. These conditions act against the pattern-forming feedback associated with overland water flow and soil-water diffusion; the infiltration of surface water into bare soil reduces overland water flow, and water uptake by laterally spread roots reduces lateral soil-water gradients and thus soil-water diffusion.

The Trindade ecosystem also exemplifies a so-far-unstudied form of patchy invasion—incomplete invasion. At precipitation rates below a characteristic threshold (*P*_inv_), the *Guilandina* invasion begins but does not spread; it rather remains in the form of isolated *Guilandina* spots of fixed size in an otherwise *Cyperus* grassland. Above this threshold, these spots generate new *Guilandina* spots in their neighborhoods, a process that repeats itself until the native species, *Cyperus*, is completely excluded. The threshold *P*_inv_ reflects an instability of a single *Guilandina* spot solution and convergence to a coexisting periodic solution through a spot-replication process.

Spot replication in other contexts usually manifests itself by the deformation of the circular spot shape followed by spot splitting. This is unlike the birth of new distant spots that is typical of *Guilandina*’s spot replication. Nevertheless, both cases share the same bifurcation structure—bistability of uniform and patterned states and an instability of a single-spot state, which implies a shift from a single-spot state to a patterned state through a process of spot replication. That universal bifurcation structure is responsible for the two forms of invasion, complete and incomplete, irrespective of the particular form of the spot replication, spot splitting, or birth of distant spots, and irrespective of the scale-dependent feedback that generates the patterned state. In the present case, *Guilandina* self-organizes in spatial patterns by the root-augmentation feedback, and new spots are born at a distance by nonlocal root suckers, but the same forms of invasion are expected with other species and soil types, where different scale-dependent feedback applies, e.g., that associated with overland water flow or soil-water diffusion, as well as modes of spatial spread, such as seed dispersal.

The effects of climate change on species invasion are attracting increasing interest, and attempts to estimate the responses of both native and invasive species and, thus, the integrated impact of climate change on the invasion process, are being made (57). Our analysis of the invasion dynamics of pattern-forming species suggests a new response form where drier climates act selectively on the invasive species, imposing an incomplete invasion and, thereby, reducing the negative effects on the native species.

## Materials and methods

### Empirical methods

#### Root distributions

We measured the distribution of roots of *Guilandina* (between 2015 December and 2016 February) at each of the four sites (Fig. S1). In sites 4 to 22, we selected one patch of *Guilandina*, the bare zone halo around it, and the *Cyperus* zone surrounding the bare zone. In site 1, where halos are absent, we selected samples in soil zones covered by *Guilandina* and *Cyperus* and in soil zones not covered by any of the species. We collected four soil samples from each of these zones. For each sample, a cylindrical hollow metal tube, 40 cm in length and 10 cm in diameter, was driven into the soil, then the soil core was removed from the tube and subdivided into 10-cm-long subsamples. Each subsample was sieved, and roots of *Guilandina* with diameter 2 mm or less were removed, dried at 50^○^C for 72 h, and weighed. We also followed and excavated dozens of large (3 to 5 cm in circumference) roots of *Guilandina* extending beyond the patches to determine if they extended into the bare-soil zones and *Cyperus* zones.

#### Soil water potential

Soil water potentials were measured at the four sites over 32 days between 2015 December and 2016 February. Sampling was conducted eight times at each of the four sites. For each day, we took measurements at 9:00 am, 12:00 pm, 2:30 pm, and 12:00 am, at depths of 4 to 5 and 9 to 10 cm in soils under *Guilandina*, in the bare soil, and in soils under *Cyperus* zones. For each site–zone–date–depth combination, we collected two replicates and analyzed them separately. Each soil sample was stored in a hermetically sealed capsule and transported to the laboratory at the Trindade Island Scientific Station, where soil water potentials were recorded with a Water Potential Meter WP4C (Decagon devices), within 12 h of soil collection. The dew point sensor inside the WP4C is the measure of water potential and is accurate to 0.05 MPa from 0 to −5 MPa and 1% from −5 to −300 MPa (58).

### Modeling root-augmentation feedback

In modeling the root-augmentation feedback, we focus on two essential elements: nonlocal water uptake by laterally spread roots and root augmentation as the shoot grows (15, 22). We model these elements by introducing a kernel function $G_i(\mathbf {X},\mathbf {X}^{\prime },T)=G_i\left[|\mathbf {X}-\mathbf {X}^{\prime }|/\mathcal {S}_{i}(B_i(\mathbf {X},T))\right]$, *i* = 1, 2, where $\mathbf {X}=(X,Y)$ are the space coordinates in the lateral directions and *T* is time. The kernel *G_i_* represents the spatial distribution of the root zone of the *i*th species that is functional in water uptake. The point in space $\mathbf {X}$ is the shoot location, and $\mathbf {X}^{\prime }$ is a distant point where uptake occurs. As $|\mathbf {X}-\mathbf {X}^{\prime }|$ increases beyond the characteristic length $\mathcal {S}_i$, which represents the lateral extension of the root zone, *G* tends to zero. The root augmentation as the shoot grows is captured by allowing *S_i_* to be a monotonically increasing function of the aboveground biomass *B_i_*.

In the present study, we consider the functional range of the roots, represented by the root kernel *G_i_*, to be of Gaussian type,

(1) $$\begin{eqnarray*}
G_i = \frac{1}{2 \pi S_{G_i}^2} \operatorname{exp}\left(-\frac{|\mathbf {X}-\mathbf {X}^{\prime }|^2}{2 \mathcal {S}_i (B_i(\mathbf {X}, T))^2}\right),~~i=1,2,
\end{eqnarray*}$$

and take $\mathcal {S}_i=S_{G_i} (1 + E_i B_i)$, where $S_{G_i}$ is the lateral root system size when the shoot emerges, and *E_i_* is the root’s augmentation per unit aboveground (shoot) biomass. We can then write the following expressions for the vegetation growth rate:

(2) $$\begin{eqnarray*}
G_{B}^{(i)} = \Lambda _i \int _\Omega G_i(\mathbf {X},\mathbf {X}^{\prime },T) W(\mathbf {X}^{\prime },T) d\mathbf {X}^{\prime }
\end{eqnarray*}$$

and water uptake rate:

(3) $$\begin{eqnarray*}
G_{W}^{(i)} = \Gamma _i \int _\Omega G_i(\mathbf {X}^{\prime },\mathbf {X},T) B_i(\mathbf {X}^{\prime },T) d\mathbf {X}^{\prime }
\end{eqnarray*}$$

of species *i*, where Λ_*i*_ is a biomass-growth coefficient per unit soil water and Γ_*i*_ is a water-uptake coefficient per unit biomass.

Finally, the root distribution of *Cyperus* is laterally confined compared to that of *Guilandina*. For both simplicity and computational efficiency, we therefore take the limit $S_{G_2} \rightarrow 0$, which gives the following local expressions for the growth and uptake rates of species 2, respectively (3):

(4) $$\begin{eqnarray*}
G_{B}^{(2)} = \Lambda _2 W_1 (1+E_2 B_2)^2,~~ G_{W}^{(2)} = \Gamma _2 B_2 (1+E_2 B_2)^2\, .
\end{eqnarray*}$$

### Modeling nonlocal sprouting

We model the new clones that emerge at a distance from the mother plant as root suckers, that is, shoots springing from the roots of the mother plant where water availability is high enough. We distinguish between root suckers and “potential root suckers”, where the latter refer to nodes along the roots that do not sprout and grow shoots because of insufficient water availability, such as in the bare-soil halos (Fig. S3 in Supplementary Material). To model the distribution of potential root suckers, we do not use the root kernel, *G*_1_ of Eq. 1, because that kernel represents the distribution of *Guilandina*’s roots that are active in water uptake for the mother plant. That distribution has a shorter range than the physical root distribution because part of the water taken up by the roots is allocated to the root suckers. On the other hand, the likelihood of finding a potential root sucker on a mother-plant’s root at a given location is significantly lower than the likelihood of solely finding that root passing at this location. We therefore model the distribution Φ_1_ of potential root suckers using a kernel with fat tails that is nevertheless much narrower than the root kernel *G*_1_. A possible choice is the Cauchy distribution (59)

(5) $$\begin{eqnarray*}
\Phi _1 = \frac{1}{2 \pi } \left[ \frac{S_{D_1}}{\left(|\mathbf {X}-\mathbf {X}^{\prime }|^2+S_{D_1}^2 \right)^{3/2}} \right]\, ,
\end{eqnarray*}$$

where the width of the distribution, as quantified by the parameter $S_{D_1}$, is typically much smaller than $\mathcal {S}_1(B_1)$ for grown plants (*B*_1_ > 0). Other choices of fat-tail distributions that yield nonlocal sprouting are possible and therefore the particular form of the distribution is of lesser significance.

We now model the growth rate, $\mathcal {D}_1$, of a *Guilandina*’s root sucker at a point $\mathbf {X}$ by

(6) $$\begin{eqnarray*}
\mathcal {D}_1 = \Theta _1 \frac{W(\mathbf {X},T)}{W(\mathbf {X},T)+W^{*}_1}\int _\Omega \Phi _1(|\mathbf {X}-\mathbf {X}^{\prime }|) B_1(\mathbf {X}^{\prime },T) d\mathbf {X}^{\prime }\,.
\end{eqnarray*}$$

In these equations, Θ_1_ represents the potential root-sucker growth rate (in units of 1/y) in the absence of water limitation, and $W_1^{*}$ is the soil-water content at which the root sucker exploits half of its growth potential. Note that the growth rate, $\mathcal {D}_1$, depends on water availability (60), which increases outside the dense range of the root zone (see Fig. S3 in Supplementary Material), and thus favors the emergence of root suckers sufficiently far from the mother plant.

### Full model equations

The high infiltration rates of surface water into the soil (see Patch-scale observations) suggest that overland water flow is negligible and allows the elimination of the equation for the surface water *H* as described in earlier studies (3, 22). We are then left with the following three equations for the aboveground biomass densities of *Guilandina, B*_1_, and *Cyperus, B*_2_, and for the soil-water content, *W*, where all variables are in units of kg/m^2^, and are functions of space $\mathbf {X}=(X,Y)$ and time *T* in units of meters and years, respectively:

(7a) $$\begin{eqnarray*}
\frac{\partial B_i}{\partial T} &=& G_{B}^{(i)}(B_i,W) B_i \left( 1 - B_i/K_i \right) - M_i B_i + \mathcal {D}_i(B_i,W), \nonumber \\
&&\qquad i = 1, 2,
\end{eqnarray*}$$

(7b) $$\begin{eqnarray*}
\frac{\partial W}{\partial T} = P - L(B_1,B_2) W - W\sum _i G_W^i(B_i)+ D_{W} \Delta W \, ,
\end{eqnarray*}$$

where Δ = ∂^2^/∂*X*^2^ + ∂^2^/∂*Y*^2^. In Eq. 7a, for simplicity, $\mathcal {D}_2$ is assumed to have the same functional form as $\mathcal {D}_1$, except that in this case it represents a dispersal kernel with a short dispersal range, $S_{D_2}$, of *Cyperus* seeds (60). The particular choice of this kernel has an insignificant effect on the results. The parameters *K_i_* in this equation represent growth limitations not associated with water availability, such as self-shading (61), and the parameters *M_i_* represent rate reduction of aboveground biomass growth due to mortality, resource allocation to reproduction, etc. In Eq. 7b, *P* is the precipitation rate, *D_W_* is the soil-water diffusion rate, and *L* is a biomass-dependent soil-water evaporation rate,

(8) $$\begin{eqnarray*}
L=N \left( 1 - \sum _i R_i B_i(\mathbf {X},T)/K_i\, \right).
\end{eqnarray*}$$

In this expression, *N* is the evaporation rate in bare soil and the parameters *R_i_* ≪ 1 (*i* = 1, 2) quantify the contributions of the two species to the reduction of the evaporation rate by shading.

A description of all model parameters, their units, and their values is given in Table 1. Deviations from these values are explicitly specified wherever they are relevant.

**Table 1.** Description of model parameters in Eq. 7.

Symbol	Description	Units	Value
*P*	Precipitation	mm/y	variable
*E_i_*	Lateral root augmentation per unit (aboveground) biomass	m^2^/kg	16 (5)
*K_i_*	Maximum standing biomass	kg/m^2^	0.7 (0.35)
*M_i_*	Biomass decay rate due to mortality and other factors	1/y	7.05 (7.05)
*N*	Evaporation rate in bare soil	1/y	15
Λ_*i*_	Coefficient of biomass-growth rate per unit soil water	(1/mm)/y	0.06 (0.16)
Γ_*i*_	Coefficient of water-uptake rate per unit aboveground biomass	(m^2^/kg)/y	15 (5)
*R_i_*	Coefficient of evaporation reduction due to shading	—	0.1 (0.1)
Θ_1_	Potential root-sucker growth rate of *Guilandina*	1/y	3.125
Θ_2_	Potential dispersal rate of *Cyperus*	1/y	3.125
*D_W_*	Lateral diffusion rate of soil moisture	m^2^/y	0.5
$S_{G_1}$	Lateral root-system size of *Guilandina*’s seedlings	m	0.5
$S_{D_1}$	Width of *Guilandina*’s potential root sucker distribution	m	0.5
$S_{D_2}$	Characteristic dispersal range of *Cyperus*	m	0.01
$W_1^{*}$	Soil-water content at half *Guilandina*’s root sucker potential growth rate	kg/m^2^	0.5
$W_2^{*}$	Soil-water content at half potential dispersal rate of *Cyperus*	kg/m^2^	2.0

These parameters capture the patchy invasion behavior observed on the low-altitude slopes of Trindade Island. Values associated with species *i* = 1 (*i* = 2) are unbracketed (bracketed).

### Model analysis

We solved the model Eq. 7 numerically by implementing a pseudo-spectral method with Runge–Kutta fourth-order time stepping [see, for instance, Ref. (62)]. This enabled us to compute diffusion and convolution terms as less expensive multiplications in Fourier space. The term $G_B^{(1)}$, however, is not a convolution due to the *B*_1_ dependence of *G*_1_, and so we used the method of Gilad et al. (63) to approximate it as a linear combination of convolutions. Gilad et al. (63) showed that this approximation can achieve a high level of accuracy using a relatively small number of convolutions. We used a linear combination of five convolution terms in our model simulations to achieve a reasonable trade-off between accuracy and computational efficiency.

The model Eq. 7a is highly nonlocal due to the inclusion of the fat-tailed dispersal kernel (5). As such, the implicitly imposed periodic boundary conditions associated with spectral methods meant that it was necessary to run invasion simulations (see Fig. 7) on an extended domain to avoid unrealistic boundary interactions with nonlocal invasion fronts. To facilitate the large computational expense of this, we implemented our spectral algorithms on a graphics processing unit (GPU) using the Python package CuPy (64), which allowed us to solve the model quickly on much larger domains.

Bifurcation diagrams were constructed by numerically continuing uniform solutions using the software package AUTO-07p (65). Computational difficulties associated with the numerical continuation of integro-partial differential equations meant that nonuniform solution branches were calculated by solving the model equations via the spectral method described previously, and not in AUTO-07p. For this reason, unstable sections of nonuniform solution branches are not included in our bifurcation diagrams. Accuracy of stable branches was achieved by allowing solutions to evolve to an asymptotic steady state; numerically, we ensured that the mean absolute error between solutions at consecutive time steps was less than some acceptable tolerance.

## Supplementary Material

pgac294_Supplemental_Files

## Data Availability

Movies showing simulations of incomplete and complete invasion are available in the Supplementary Material. Sample versions of our code along with instructions to reproduce our results have been uploaded to the GitHub repository, github.com/03bennej/trindade-2022.
