# Correction to: Evidence for scale-dependent root-augmentation feedback and its role in halting the spread of a pantropical shrub into an endemic sedge

**DOI:** 10.1093/pnasnexus/pgad116

**Published:** 2023-04-11

This is a correction to: Jamie J R Bennett, Anabele S Gomes, Michel A Ferré, Bidesh K Bera, Fabian Borghetti, Ragan M Callaway, Ehud Meron, Evidence for scale-dependent root-augmentation feedback and its role in halting the spread of a pantropical shrub into an endemic sedge, PNAS Nexus, Volume 2, Issue 1, January 2023, pgac294, https://doi.org/10.1093/pnasnexus/pgac294

In the originally published version of this manuscript, the title contained a typographical error and was given as follows:

‘Evidence for scale-dependent root-antation feedback and its role in halting the spread of a pantropical shrub into an endemic sedge’.

The title has now been corrected to:

‘Evidence for scale-dependent root-augmentation feedback and its role in halting the spread of a pantropical shrub into an endemic sedge’.

