# Supplementary material for: Evidence for scale-dependent root-augmentation feedback and its role in halting the spread of a pantropical shrub into an endemic sedge
Source: PNAS Nexus. 2022 Dec 19;2(1):pgac294. doi: 10.1093/pnasnexus/pgac294 (PMC9887939; doi:10.1093/pnasnexus/pgac294)
Supplement: pgac294_Supplemental_Files [file pgac294_supplemental_files.zip › PNASNEXUS-PNASNEXUS-2022-01117-T-s01.pdf]

Supporting Information for:

Evidence for scale-dependent root-augmentation feedback and its role in halting the spread of a pantropical shrub into an endemic sedge

Jamie J. R. Bennett[a], Anabele S. Gomes[b], Michel Ferré[a], Bidesh K. Bera[a], Fabian Borghetti[b], Ragan M. Callaway[c], Ehud Meron[a,d1]

[a]Department of Solar Energy and Environmental Physics, Blaustein Institutes for Desert Research, Ben-Gurion University of the Negev, Sede Boqer Campus 8499000, Israel; [b]Department of Botany, University of Brasília, Brasília, 70910-900, Brazil; [c]Department of Wildlife Biology, University of Montana, Missoula, MT 59812, USA; [d]Physics Department, Ben-Gurion University of the Negev, Beer Sheva 8410501, Israel

Empirical results

We present here empirical results obtained in the four study sites illustrated in Fig. 1. Across all low-altitude sites (2-4), the biomass of *Guilandina*'s fine roots beneath the bare-soil halos was relatively high, about 40% of the biomass beneath *Guilandina* as Fig. 2 (right panel) shows. This is in sharp contrast to the high-altitude site where the biomass of *Guilandina*'s fine roots beneath sporadic bare-soil areas was much smaller than the biomass beneath *Guilandina* (left panel). In the low-altitude sites *Guilandina*'s roots extend even beyond the bare-soil halos to the *Cyperus* grassland area as the nonzero biomass value there indicates. In the high-altitude site, the biomass of *Guilandina*'s fine roots in *Cyperus* areas is also nonzero but there *Guilandina* patches are adjacent to *Cyperus* patches.

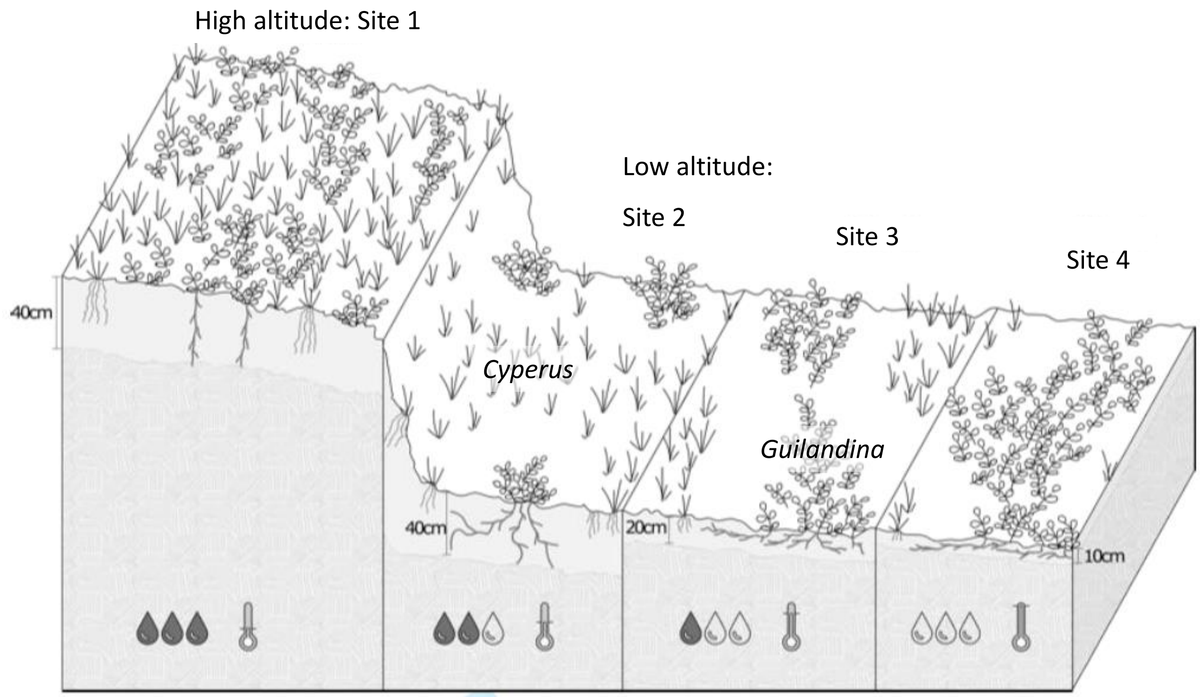

Fig. 1. Schematic illustration of the study sites on Trindade Island. Site 1 is a high-altitude site (circa 600m) whereas sites 2, 3, and 4 are low-altitude sites (circa 60-100m). The illustration provides information about estimated soil and rooting depths, and general relative gradients of precipitation and temperature.

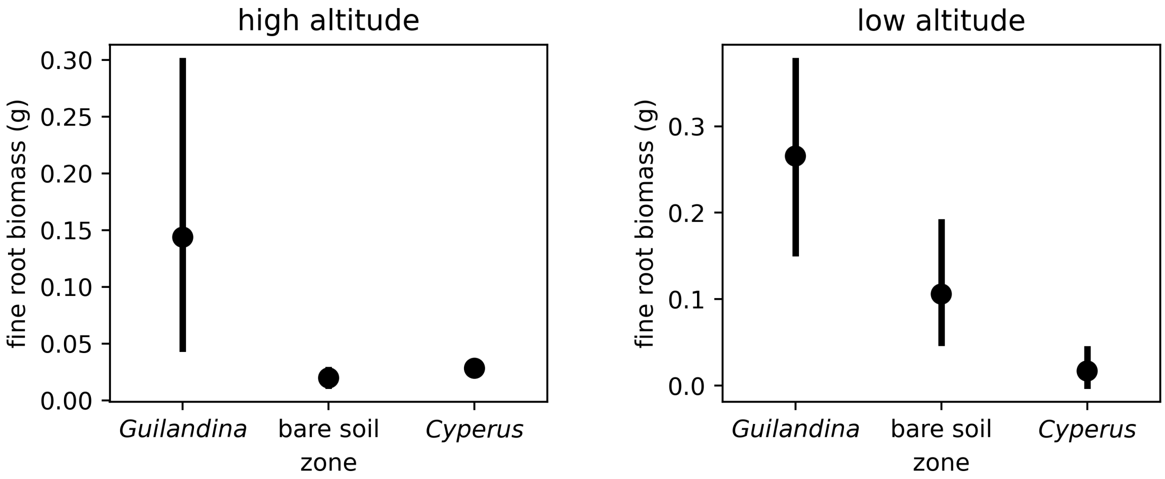

Fig. 2. Average dry biomass (g root / 3140cm^3 soil) of fine roots of *Guilandina bonduc* in soil samples collected in high-altitude (left) and low-altitude (right) sites under *Guilandina*, bare soil and *Cyperus* zones at 40 cm deep on the Trindade Island, Brazil. The bars represent 95% confidence intervals.

Soil-water potentials in the low-altitude sites were much lower in bare zones than in either the *Cyperus* or the *Guilandina* zones, especially at the shallower measurements at the soil depth of 5 cm. as Fig. 3 shows. The lowest value of a single soil-water potential measurement was -4.8 MPa in a bare-soil halo of a *Guilandina* patch. Soil-water potentials at the high-altitude site were much higher than those of the low-altitude sites across all zones.

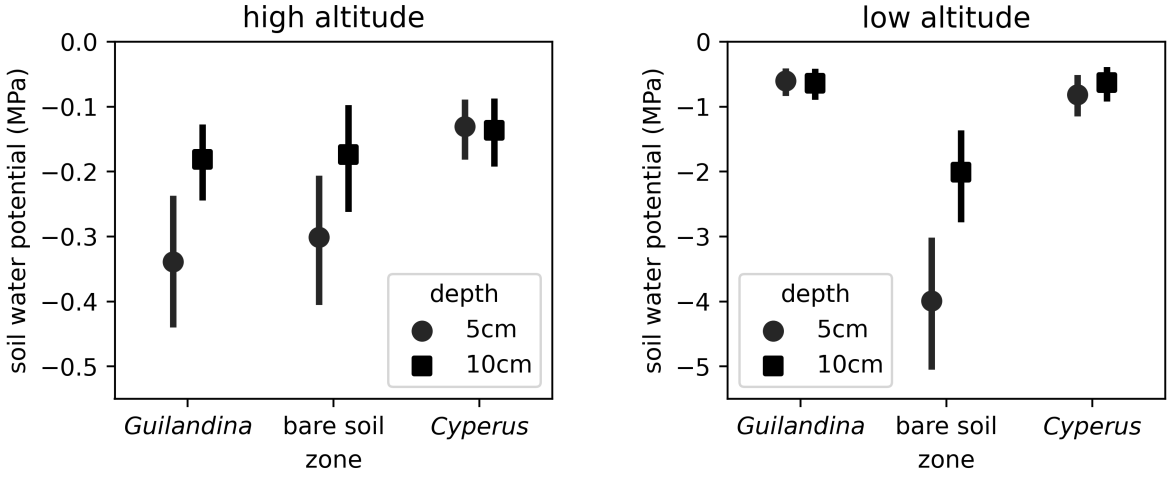

Fig. 3. Average soil-water potential (MPa) of soil samples collected at different soil depths (5 and 10 cm) on soils covered by monodominant stands of *Guilandina bonduc*, of *Cyperus atlanticus* and in bare soil zones. Shown are average values for the high-altitude site (left) and for the low-altitude sites (right). The bars represent 95% confidence intervals.

Numerical code to solve the model equations

The reader is referred to the GitHub repository github.com/03bennej/trindade-2022 for information about the GPU code to solve the integro-partial differential equations of the model presented in the main text (Eqs. (7)). The model equations that are solved are a non-dimensional version of Eqs. (7) and are given in the above GitHub repository, along with the definitions of the non-dimensional variables and parameters.

SI Movies

Movie M1. Movie showing incomplete patchy invasion as seen in the top part of Fig. 8A, resulting in coexistence of the native and invasive species. Initial small *Guilandina* patches expand, but below a critical threshold value of mean annual precipitation, $P = P_{inv}$, water stress prevents further patch expansion. Isolated *Guilandina* patches form circular spots of characteristic fixed

size surrounded by bare-soil halos in otherwise uniform *Cyperus* grassland. The circular shape is deformed when the initial patches are close to each other and interact.

Movie M2. Movie showing complete patchy invasion as seen in the bottom part of Fig. 8A, resulting in complete exclusion of the native species. Initial small *Guilandina* patches expand and approach a characteristic size, but above P_{inv} they initiate the appearance of new distant *Guilandina* patches, and trigger a spot-replication process that results in *Guilandina*-only patchy landscape.
